# A Manual of Minor Surgery and Bandaging

**Published:** 1875-11

**Authors:** 


A Manual of Minor Surgery and Bandaging. By Christopher Heath, F.R.C.S. Fifth edition. Published by Lindsay and Blakiston, Philadelphia. Book; cloth; pp. 302.

This work is specially devoted to the management and treatment of wounds, with the means of reducing dislocations, etc. The various bandages and their mode of application are ably and practically presented by numerous illustrations and woods cuts. It is a work which should be in the hands of physicians, especially the young, when they need something to which they can refer in cases of accidents and emergencies. It also teaches the manner of performing many of the most trying surgical operations, and other subjects of practical importance.

